# Prevalence of symptoms of anxiety and depression one year after intensive care unit admission for COVID-19

**DOI:** 10.1186/s12888-024-05603-8

**Published:** 2024-03-01

**Authors:** Netha Hussain, Carina M. Samuelsson, Avril Drummond, Carina U. Persson

**Affiliations:** 1 Department of Radiology, Sahlgrenska University Hospital, Region Västra Götaland, Gothenburg, Sweden; 2 Department of Occupational Therapy and Physiotherapy, Sahlgrenska University Hospital/Östra, Region Västra Götaland, Gothenburg, Sweden; 3 School of Health Sciences, University of Nottingham, Nottingham, UK; 4 Department of Clinical Neuroscience, Rehabilitation Medicine, Institute of Neurosicence and Physiology, Sahlgrenska Academy, University of Gothenburg, Göteborg, Sweden

**Keywords:** Anxiety, Depression, COVID-19, Intensive care unit, Patient reported outcome measure

## Abstract

**Background:**

To the best of our knowledge, the long term prevalence of symptoms of anxiety and depression in ICU admitted individuals after COVID-19 in Sweden during the first wave of the pandemic has not been investigated. Furthermore, no studies have exclusively investigated the risk factors for experiencing symptoms of anxiety and depression in this population.

**Aim:**

The aim of this study was to determine the prevalence of symptoms of anxiety and depression at one year after ICU admission for COVID-19. An additional aim was to identify any early predictors that are associated with symptoms of anxiety and depression, at one year following ICU admission for COVID-19.

**Methods:**

This multicenter cohort study had a cross-sectional and a longitudinal design. The primary outcomes and dependent variables, symptoms of anxiety and depression, were assessed using the Hospital Anxiety and Depression Scale (HADS). The independent variables were related to demographic factors, comorbidities, and complications during COVID-19-related ICU admission. Logistic regression analyses were performed to identify any predictors of symptoms of anxiety and depression.

**Results:**

Out of 182 eligible individuals, 105 participated in the study. Symptoms of anxiety was found in 40 (38.1%) and depression in 37 (35.2%) of the participants. Using univariable logistic regression analyses, female sex was identified as a predictor of depression as defined by HADS at one year following ICU admission for COVID-19 (odds ratio 2.53, 95% confidence intervals 1.01–6.34, *p*-value 0.048).

**Conclusions:**

The high prevalence of symptoms of anxiety and depression in ICU admitted individuals one year after COVID-19 is a public health issue of concern. Our findings imply that individuals who recovered after an ICU stay for COVID-19 may benefit from long-term follow-ups and continuous mental health support for more than a year following the ICU admission. For women specifically, this is true.

**Trial registration:**

The study was registered at researchweb.org on 28 May 2020 (Project number: 274477).

**Supplementary Information:**

The online version contains supplementary material available at 10.1186/s12888-024-05603-8.

## Introduction

The COVID-19 pandemic resulted in an unprecedented surge of individuals requiring treatment at the Intensive Care Unit (ICU), particularly during the first wave of the pandemic [1]. Although COVID-19 ceased to remain a public health emergency of international concern [2], the number of ICU survivors have naturally increased since the start of the pandemic, which is why it is important to understand more about the long-term effects related to COVID-19.

Evidence suggesting the presence of long-term mental health consequences in ICU admitted individuals after COVID-19 are starting to take shape [3–9]. At three months following ICU admission for COVID-19, 23% and 29% reported anxiety and depression, respectively [5], and ICU survivors of COVID-19 were found to have higher mean depression than the matched general population [6]. At five months after ICU admission for COVID-19, a Swedish study reported 33% and 36% anxiety and depression, respectively [7]. A Dutch study showed that 17.9% and 18.3% of ICU survivors of COVID-19 had anxiety and depression, respectively, at one year follow-up [8]. Similarly, at one year follow-up, a Brazilian study with 1,156 participants showed that those who received mechanical ventilation for COVID-19 were found to experience higher anxiety than those who did not [9]. Due to its high prevalence and long-lasting consequences, country-specific prevalence of anxiety and depression after COVID-19 are important to investigate from a public health perspective.

To the best of our knowledge, no studies have exclusively investigated the risk factors of symptoms of anxiety and depression in ICU admitted individuals one-year after COVID-19. Knowledge regarding the risk factors of symptoms of anxiety and depression in ICU admitted individuals for COVID-19 will allow for providing individualized care for at-risk individuals. Therefore, the aim of this study was to determine the prevalence of symptoms of anxiety and depression at one year after ICU admission for COVID-19. An additional aim was to identify any early predictors prior to and during the care period at ICU that are associated with symptoms of anxiety and depression at one year following ICU admission for COVID-19. Based on clinical reasoning and similar studies from previous research based on hospitalized patients due to COVID-19, we hypothesized that symptoms of anxiety and depression at one year following ICU admission for COVID-19 was associated with age [10], sex [11], and length of stay at the ICU [10], along with co-morbidities [12] and complications that arose during the ICU stay period.

## Methods

### Study design

**Fig. 1:**
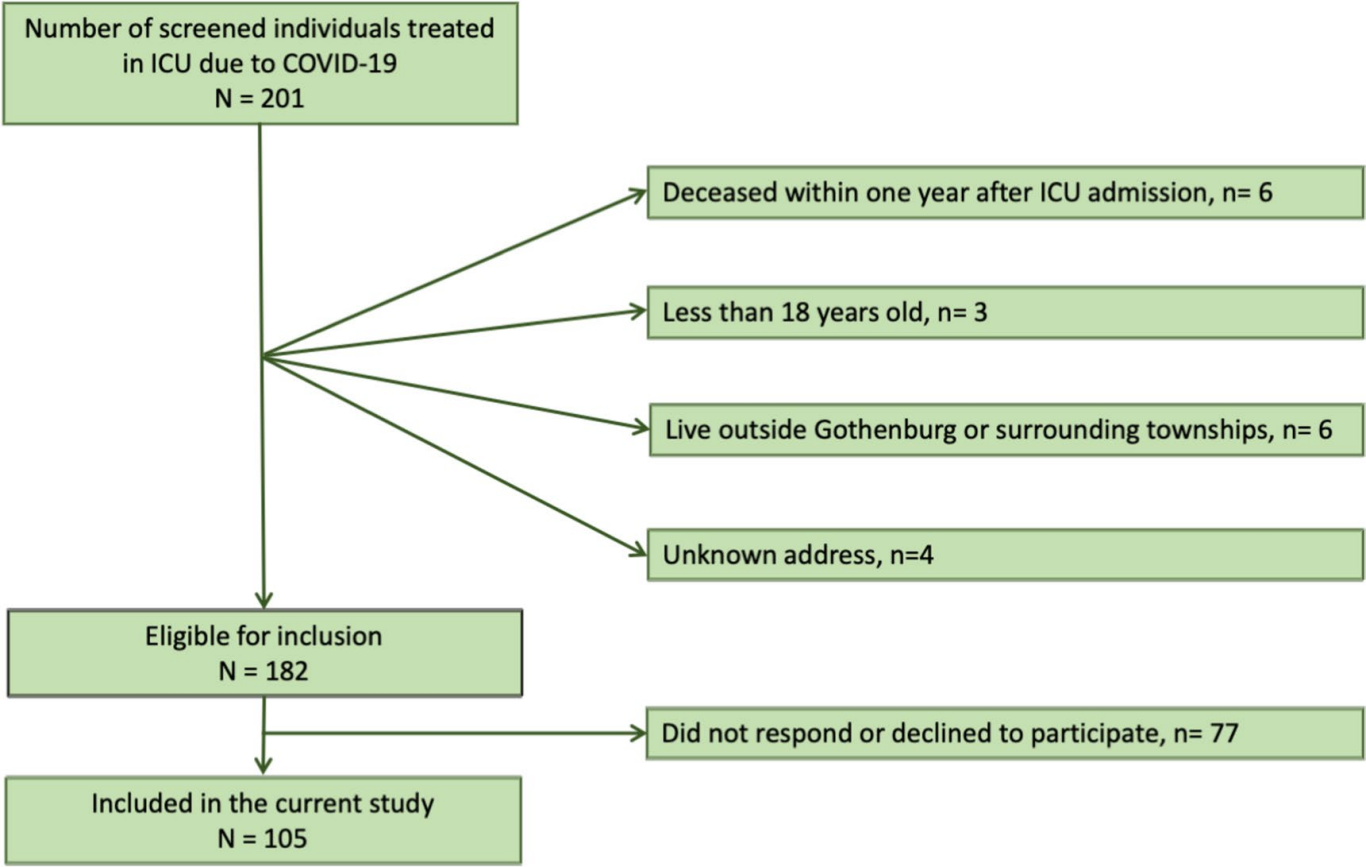
Flow chart of the participant recruitment

This multicenter cohort study, the Gothenburg Recovery and Rehabilitation after COVID-19 and Intensive Care Unit (GOT-RECOV-19 ICU), had a cross-sectional and longitudinal observational design. The outcome measures were collected at a one-year follow-up after COVID-19. The GOT-RECOV-19 ICU was registered at “FoU i Sverige” (researchweb.org), the open project database for research projects from Sweden, on May 28, 2020 (ID number: 274477). Individuals aged 18 or older who were admitted to any of the five ICUs at Sahlgrenska University Hospital/Östra between 2020-03-01 and 2020-06-30 with the diagnostic code UO7.1 (COVID-19 virus detected, according to classification system ICD-10 SE) and were still alive one year later met the inclusion criteria. The exclusion criteria were individuals not registered as a resident of Sweden in the Swedish Population Register or not living in Gothenburg or its surrounding municipalities (Fig. 1).

### Procedure

The data collection for GOT-RECOV-19 ICU started in March 2021, when patient record data for individuals admitted to the ICU with COVID-19 as the primary diagnosis was collected from the Data Output Unit at the Sahlgrenska University Hospital. We identified 259 participants by means of their social security number and found that 58 had died before the one-year follow-up and 19 did not meet the inclusion criteria. The remaining eligible 182 potential participants were sent a postal invitation for participation in the study. This was followed up with, at most, two reminders in case the earlier invitations were not received. In the second reminder invitation, the potential participants were given an option to respond exclusively to the questionnaires postally or digitally, without having to participate in the clinical assessments. The inclusion criteria are shown in Fig. 1.

### Data collection of the potential predictors at the baseline

For those who consented to participate in the study, age, sex, length of ICU stay, presence of relevant comorbidities and complications during ICU stay, were obtained from the medical records. This information was based on the consulting doctor’s assessment of the individual on their admission to the ICU. Key comorbidities collected were diabetes mellitus, hypertension, coronary heart disease, asthma, chronic kidney disease, chronic heart failure and chronic obstructive pulmonary disease (COPD) [yes/no]. The complications during ICU care considered for this study were sepsis and acute respiratory distress syndrome (ARDS) [yes/no]. Surgical Procedure Assessment Score (SPA) score was unfortunately not available as it is not routinely performed in Sweden. Similarly, SPA score is performed on individuals on ventilator, while our study included participants who were both on and off ventilator. We have chosen to use the length of stay at the ICU as a proxy indicator for the severity of the disease instead of time in the ventilator, and we assume that these variables are likely to show a high degree of correlation with each other – which was why only one of these were chosen.

### Data collection of the primary outcomes at the one-year follow-up

Starting in March 2021, the primary outcomes, symptoms of anxiety, and depression, were assessed using the Hospital Anxiety and Depression Scale (HADS) [13, 14]. The HADS comprises seven questions each for anxiety and depression and takes two-five minutes to complete [15]. Scoring for each item ranges from zero to three, with three denoting highest anxiety or depression level. A total score in the anxiety and the depression subscales between 8 and 10 is considered as mild, 11–14 as moderate and 15–21 as severe anxiety or depression, respectively [15]. The psychometric properties of the HADS have been assessed in hospitalized individuals for COVID-19, where it was found to have good construct validity and internal consistency [16]. Additionally, the Swedish language version of HADS [17], used in the current study, has been previously evaluated in a Swedish population and was therefore thought to be a valid clinical tool to indicate the symptoms of anxiety and depression [18]. However, although HADS can be used as a screening tool for the symptoms of anxiety and depression; and cannot be used for diagnosis of these conditions.

All data collection for the baseline and outcome variables was performed by two physiotherapists and researchers (CMS and CUP), both of whom are co-authors of this study. For the participants who performed the assessments onsite at Sahlgrenska University Hospital/Östra, Gothenburg, Sweden, HADS was performed as the seventh outcome measure in the order of the battery of assessments included in the GOT-RECOV-19 ICU study.

### Statistical analyses

The statistical analyses were performed using the IBM Statistical Package for Social Sciences (SPSS) software version 28 and the images were created using PowerPoint on Microsoft 365 version 16.2 and Adobe Photoshop version 20. The demographic characteristics (related to age, sex and length of stay) between those who participated in the study and those who declined to participate/did not respond to the study request were performed using the Mann–Whitney U-test. A sub-analysis using Mann–Whitney U-test was conducted to check if there were any significant differences in HADS score between those who responded to the questionnaire during the different seasons of the year [19]. The level of significance was set to *p* < 0.05 (two-tailed).

Descriptive statistics, such as absolute numbers, percentages, means, standard deviations (SDs), medians, and interquartile ranges (IQRs) were presented, showing the demographic characteristics, prevalence of comorbidities and complications as well as HADS score. The HADS scores for both anxiety and depression were dichotomized into two groups as ‘no anxiety/depression’ or ‘anxiety/depression present’, with ≥ 8 indicating presence of anxiety/depression. In case of missing data, interval estimation was performed in suitable cases.

First, univariable binary logistic regression analyses were performed with dichotomized HADS scores of anxiety and depression, respectively, as the dependent variable, and age, sex, length of ICU stay, diabetes mellitus, hypertension, heart disease, ARDS, and sepsis as the independent variables. The ethical approval for our study allowed us to obtain extracts of the medical records of the participants during the time they were admitted in the ICU. Therefore, only the comorbidities collected by the ICU personnel could be used in this study. Using logistic regression analyses, the number of potential predictors was adjusted to the sample size. Given the existing population size, we decided to prioritize well-known major comorbidities such as diabetes and hypertension over less frequent ones.

The presence of asthma and COPD was so low that we considered them as lacking adequate power for including them in the regression model. Length of stay was used as a proxy for the severity of the disease. The Surgical Procedure Assessment score (SPAS) was not available as it was (and is still) not routinely performed in Sweden [20]. However, it was also irrelevant since not everyone in our population was treated with a ventilator.

Thereafter, variables in the univariable analyses with *p*-values of < 0.1 were included in the multivariable regression model. Any multicollinearity between the statistically significant independent variables was determined using the Spearman’s rank correlation, where correlation coefficients of ≥ 0.7 were considered as multicollinear [21]. Thereafter, in the multivariable regression model, a *p*-value of < 0.05 was considered as statistically significant for each independent variable. The results of the regression analyses were presented as odds ratios (ORs), 95% confidence intervals (CIs) and *p*-values. A post-hoc analysis using Mann-Whitney U-test was performed to find if any significant differences existed between females and males in terms of length of stay at the ICU.

## Results

Of the 182 individuals who were eligible for the study, 105 (57.7%) completed the HADS. Of these, 78 participants responded during their visit to the hospital, 26 responded postally and one responded digitally. The flowchart showing the participant selection for this study is shown in Fig. 1.

No statistically significant differences in terms of age (Z=-1.23, *p* = 0.22), sex (Z=-0.72, *p* = 0.47), or length of stay at the ICU (Z=-1.40, *p* = 0.16) were found between the 105 participants and the 77 individuals who declined to participate or did not respond to the study request. Similarly, no statistically significant differences in total HADS scores were found between those who responded to the questionnaire during spring or summer (Z=-0.78, *p* = 0.43), the two seasons during which the study questionnaires were sent in. The demographic characteristics, relevant comorbidities, complications during the ICU and other baseline characteristics of the study group (*n* = 105) have already been presented [22]. The demographic data for individuals with and without symptoms of anxiety/depression are shown in Table 1.

**Table 1:** Demographic characteristics of participants admitted with COVID-19, on admission to one of the five intensive care units

	All participants [Mean ± SD, Median (IQR), n (%)]			
	Anxiety subscale (*N* = 105)		Depression subscale (*N* = 105)	
	No anxiety (*n* = 65)	Anxiety present (*n* = 40)	No depression (*n* = 68)	Depression present (*n* = 37)
Age, years	59.57 ± 11.17	55.92 ± 12.65	59.41 ± 11.65	55.91 ± 12.00
Female	13 (20.0)	12 (30.0)	12 (17.6)	13 (35.1)
Male	52 (80.0)	28 (70.0)	56 (82.4)	24 (64.9)
BMI, kg/m^2^, (*n* = 54)	28.75 ± 8.03 (*n* = 34)	31.36 ± 7.51 (*n* = 21)	29.75 ± 8.78 (*n* = 37)	29.73 ± 5.79 (*n* = 18)
Length of stay in ICU, in days	13 (7–25)	18.5 (8.25–27.75)	16 (8-27.5)	14 (7–26)
*Type of respiratory support*				
Mask or nasal cannula	2 (3.1)	2 (5.0)	1 (1.5)	3 (8.1)
High Flow Oxygen Therapy	9 (13.8)	6 (15.0)	11 (16.2)	4 (10.8)
Mechanical ventilation	54 (83.1)	32 (80.0)	56 (82.4)	30 (81.1)
*Comorbidities*				
Multimorbidity^a^	26 (40.0)	16 (40.0)	30 (44.1)	12 (32.4)
Diabetes mellitus	15 (23.1)	8 (20.0)	15 (22.1)	8 (21.6)
Hypertension	27 (41.5)	17 (42.5)	29 (42.6)	15 (40.5)
Coronary heart disease	16 (24.6)	11 (27.5)	21 (30.9)	6 (16.2)
Asthma	4 (6.2)	4 (10.0)	5 (7.4)	3 (8.1)
Chronic kidney disease	5 (7.7)	1 (2.5)	5 (7.4)	1 (2.7)
Chronic heart failure	1 (1.5)	3 (7.5)	3 (4.4)	1 (2.7)
Chronic obstructive pulmonary disease	1 (1.5)	1 (2.5)	1 (1.5)	1 (2.7)
*Complications during ICU stay*				
ARDS	37 (56.9)	19 (47.5)	37 (54.4)	19 (51.4)
Sepsis	31 (47.7)	24 (60.0)	35 (51.5)	20 (54.1)

^a^Multimorbidity refers to the co-occurrence of two or more comorbidities

*Abbreviations*: *SD *Standard deviation, *IQR *Interquartile range, *BMI *Body mass index, *ICU *Intensive Care Unit, *ARDS *Acute Respiratory Distress Syndrome

Approximately three in four individuals were younger than 65 years of age. The most reported comorbidity was hypertension (42.0%) followed by coronary heart disease (26.7%) and diabetes mellitus (21.9%).

For one participant who did not score one item related to symptoms of depression, interval estimation was performed for dichotomization of the HADS score. According to HADS, nearly two in five individuals were classified to have symptoms of anxiety at one year following ICU admission for COVID-19. Furthermore, nearly one in four individuals classified as having symptoms of mild anxiety, while one in ten had symptoms of moderate anxiety. Severe symptoms of anxiety were found in 5% of the participants. The univariable regression analyses showed no statistically significant predictors of symptoms of anxiety as the dependent variable.

**Fig. 2:**
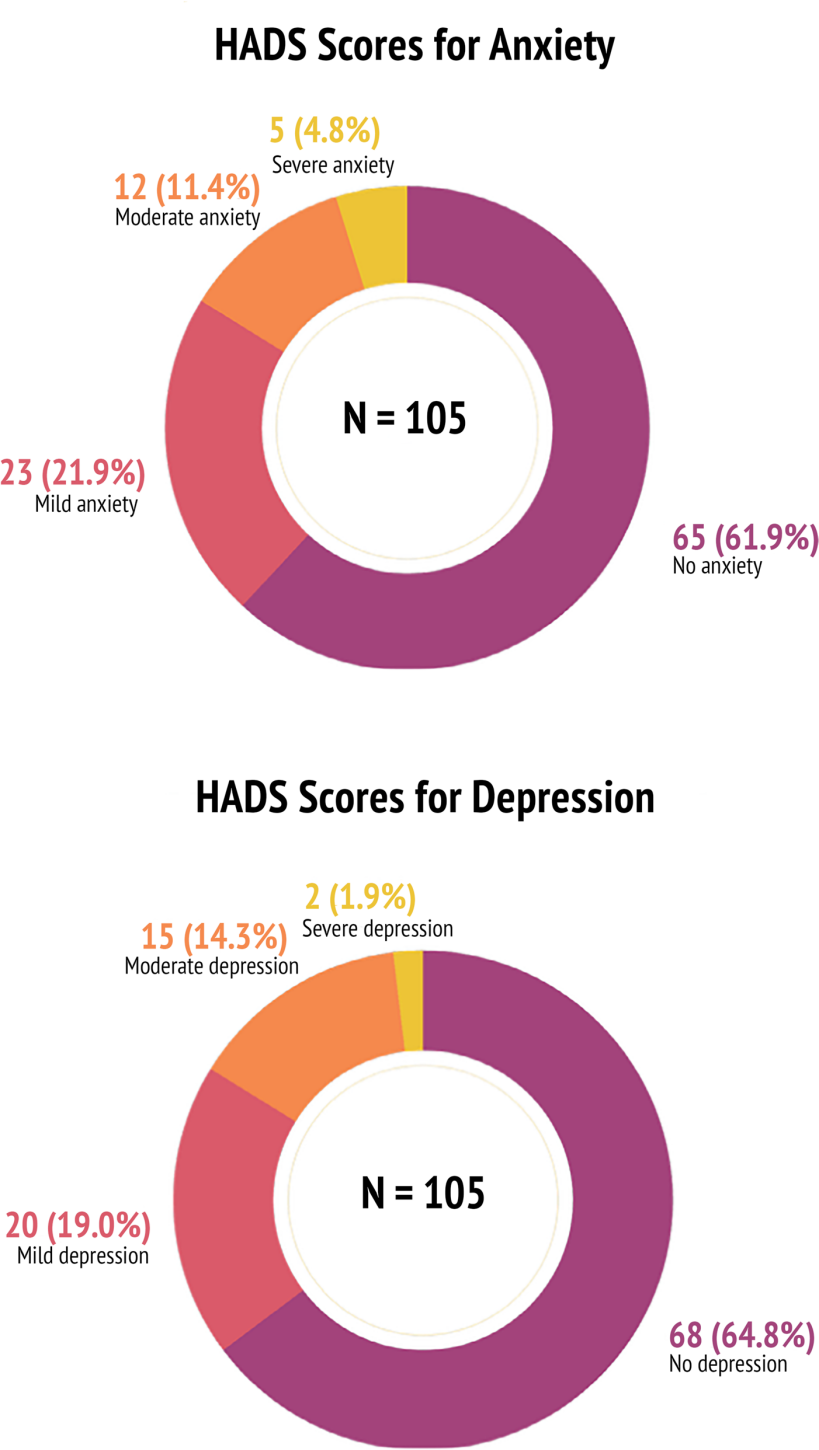
The proportion of participants showing symptoms of anxiety and depression levels as assessed using the Hospital Anxiety and Depression Scale (HADS)

 Approximately one in three individuals were classified to have symptoms of depression according to HADS at one year following ICU admission for COVID-19. One in five participants reported symptoms of mild depression. Approximately 15% of the participants were found to have symptoms of moderate depression and 2% were found to have symptoms of severe depression as assessed using the HADS. Female sex was found to be a significant predictor of symptoms of depression as assessed using dichotomized HADS score. Figure 2 shows the prevalence of symptoms of anxiety and depression, respectively.

The results of the univariable regression analyses with symptoms of anxiety and depression as the dependent variables can be found in Table 2.

**Table 2:** Results of univariable logistic regression analyses with symptoms of anxiety and depression respectively, as the dependent variables

Independent variables	Dependent variable: Anxiety		Dependent variable: Depression	
	Odds ratio (95% confidence interval)	Nagelkerke pseudo R^2^	Odds ratio (95% confidence interval)	Nagelkerke pseudo R^2^
Age	0.97 (0.94–1.01)	0.031	0.98 (0.94–1.01)	0.027
Sex (ref = male)	1.71 (0.69–4.26)	0.017	**2.53 (1.01–6.34)**	0.050
Length of ICU stay	1.00 (0.98–1.02)	0.000	0.99 (0.97–1.01)	0.005
Diabetes mellitus	0.83 (0.32–2.19)	0.002	0.98 (0.37–2.57)	0.000
Hypertension	1.04 (0.47–2.31)	0.011	0.92 (0.41–2.07)	0.011
Heart disease	1.16 (0.48–2.84)	0.001	0.43 (0.16–1.19)	0.000
ARDS	0.69 (0.31–1.51)	0.011	0.88 (0.40–1.97)	0.001
Sepsis	1.65 (0.74–3.66)	0.019	1.11 (0.50–2.48)	0.001

*Abbreviations*: *ICU *Intensive Care Unit, *ARDS *Acute Respiratory Distress Syndrome. Extended results of univariable logistic regression analyses are given in Supplementary Table 1 (Anxiety) and Supplementary Table 2 (Depression)

Bold letters indicate statistically significant odds ratio

No multivariable regression analyses were performed because no statistically significant variable emerged from the univariable regression analyses related to symptoms of anxiety, and only one statistically significant variable emerged from the univariable regression analyses for depression. Post hoc analysis showed statistically significant differences (*p*<0.001) between length of stay at the ICU between males and females.

## Discussion

In this one-year follow-up, nearly two in five participants were classified as having anxiety and roughly a third were classified to have depression according to the HADS, at one year after COVID-19. Our hypothesis that female sex was a predictor of depression was confirmed. However, our hypotheses that age, length of ICU stay, presence of some relevant comorbidities (diabetes mellitus, hypertension, heart disease, ARDS, sepsis) and presence of complications during ICU stay were predictors of anxiety and depression at one year after ICU admission could not be confirmed.

The prevalence of symptoms of anxiety (38.1%) and depression (35.2%) in this study are in line with a one-year follow up study on quality of life assessment, where 29% of those who required mechanical ventilation for COVID-19 self-reported to have either anxiety or depression [3]. However, the prevalence from our study was higher than that of a Dutch study including ICU admitted individuals for COVID-19 between March-June 2020 which showed that 17.9% and 18.3% had anxiety and depression respectively at one year follow-up [8]. These differences in the findings could be attributed to differences in pre-COVID-19 mental health status of the cohorts, cultural differences between the two countries, and the nature of follow-up and continued healthcare support after COVID-19, although these attributes are not assessed in either study. A Swedish study examining anxiety and depression at five months following ICU admission for COVID-19 showed 33% and 36% anxiety and depression respectively [7], which is nearly similar to that of the current study.

The results of our study that show that female sex was a predictor of depression at one year following ICU admission for COVID-19, is in line with another one-year follow-up study of 200 participants, though it was based on individuals hospitalized for COVID-19, not specifically cared for at an ICU [11]. Females are found to have longer, and more severe post-COVID-19 syndrome and higher proportion of depression compared to males [23, 24]. Therefore, we speculate that our results are influenced by females being disproportionately affected by post-COVID syndrome, pre-existing depression, post-ICU syndrome or inadequate rehabilitation within the year after onset of COVID-19, in a way that makes them more depressed. Furthermore, cultural norms, gender-based biases and expectations and hormonal changes in women of the perimenopausal age group might have contributed to females showing more depression after ICU recovery for COVID-19. Considering all these factors, it is not surprising that being female is a risk factor for depression among ICU admitted individuals at one year after COVID-19.

The strength of this study is the well-defined cohort of individuals from Sweden’s second highly populated county, who survived severe COVID-19 during the first wave of the pandemic. To our knowledge, this is the first study that examines risk factors for symptoms of anxiety and depression of ICU-admitted individuals at one year following COVID-19. However, this study has several limitations. The patient-reported outcome measure used in this study is a screening tool, which cannot be used to diagnose either anxiety or depression. Finally, as the onset of COVID-19 came without warning, data related to pre-COVID-19 symptoms of anxiety and depression were not collected, and to avoid recall bias, we decided not to collect data on this retrospectively. In the absence of a control group, it is not possible to determine if the prevalence and the identified risk factor of symptoms of anxiety and depression were due to COVID-19 or ICU admission or both, or de facto being in a pandemic. However, studies from the general population in Sweden, published in 2020 and 2023, show lower proportions of people having symptoms of anxiety (9.5-24.2%), and symptoms of depression (15.6-30%) [25, 26] compared with the current population of survivors of severe COVID-19.

## Conclusions

Symptoms of anxiety were present in 38.1% and symptoms of depression in 35.2% of the individuals at one year following ICU admission for COVID-19 according to the Hospital Anxiety Depression Scale (HADS). Female sex was a predictor of symptoms of depression at one year follow-up for ICU admission after COVID-19. The high prevalence of symptoms of anxiety and depression in ICU admitted individuals one year after COVID-19 is a public health issue of concern. Our findings imply that individuals who recovered after an ICU stay for COVID-19 may benefit from long-term follow-ups and continuous mental health support for more than a year following the ICU admission. This is especially true for women. 

### Supplementary Information

**Supplementary Material 1.**

## Data Availability

The dataset is available from the principal investigator, Carina U. Persson (carina.persson@vgregion.se), in response to a reasonable request. According to Swedish regulations, permission to use data can be obtained after an application to and approval by the Swedish Ethical Review Authority.
