# Supplementary material for: Prevalence of symptoms of anxiety and depression one year after intensive care unit admission for COVID-19
Source: BMC Psychiatry. 2024 Mar 1;24:170. doi: 10.1186/s12888-024-05603-8 (PMC10905917; doi:10.1186/s12888-024-05603-8)
Supplement: Supplementary file 1 — Supplementary Material 1. [file 12888_2024_5603_MOESM1_ESM.docx]

**Supplementary Table 1** Extended results of univariable logistic regression analyses with symptoms of anxiety as the

dependent variable.

| **Independent variables** | **Dependent variable:**  **Anxiety** | | | | | |
| --- | --- | --- | --- | --- | --- | --- |
|  | **Odds ratio (95% confidence interval)** | **Nagelkerke pseudo R^2^** | **B (Beta)** | **Standard Error** | **Wald** | **Degrees of freedom** |
| Age | 0.97 (0.94-1.01) | 0.031 | -0.03 | 0.02 | 2.32 | 1 |
| Sex (ref=male) | 1.71 (0.69-4.26) | 0.017 | 0.54 | 0.46 | 1.35 | 1 |
| Length of ICU stay | 1.00 (0.98-1.02) | 0.000 | 0.001 | 0.01 | 0.01 | 1 |
| Diabetes mellitus | 0.83 (0.32-2.19) | 0.002 | -0.18 | 0.49 | 0.14 | 1 |
| Hypertension | 1.04 (0.47-2.31) | 0.011 | 0.04 | 0.41 | 0.01 | 1 |
| Heart disease | 1.16 (0.48-2.84) | 0.001 | 0.15 | 0.46 | 0.11 | 1 |
| ARDS | 0.69 (0.31-1.51) | 0.011 | -0.38 | 0.40 | 0.88 | 1 |
| Sepsis | 1.65 (0.74-3.66) | 0.019 | 0.50 | 0.41 | 1.50 | 1 |

**Supplementary Table 2** Extended results of univariable logistic regression analyses with symptoms of depression as

the dependent variable.

| **Independent variables** | **Dependent variable:**  **Depression** | | | | | |
| --- | --- | --- | --- | --- | --- | --- |
|  | **Odds ratio (95% confidence interval)** | **Nagelkerke pseudo R^2^** | **B (Beta)** | **Standard Error** | **Wald** | **Degrees of freedom** |
| Age | 0.98 (0.94-1.01) | 0.027 | -0.03 | 0.02 | 2.06 | 1 |
| Sex (ref=male) | 2.53 (1.01-6.34) | 0.050 | 0.93 | 0.47 | 3.91 | 1 |
| Length of ICU stay | 0.99 (0.97-1.01) | 0.005 | -0.01 | 0.01 | 0.36 | 1 |
| Diabetes mellitus | 0.98 (0.37-2.57) | 0.000 | -0.03 | 0.50 | 0.00 | 1 |
| Hypertension | 0.92 (0.41-2.07) | 0.011 | -0.09 | 0.42 | 0.04 | 1 |
| Heart disease | 0.43 (0.16-1.19) | 0.000 | -0.84 | 0.52 | 2.61 | 1 |
| ARDS | 0.88 (0.40-1.97) | 0.001 | -0.12 | 0.41 | 0.09 | 1 |
| Sepsis | 1.11 (0.50-2.48) | 0.001 | 0.10 | 0.41 | 0.06 | 1 |
